# LEAP2, a ghrelin receptor inverse agonist, and its effect on alcohol-related responses in rodents

**DOI:** 10.1038/s41398-024-03136-y

**Published:** 2024-10-02

**Authors:** Maximilian Tufvesson-Alm, Cajsa Aranäs, Sebastian Blid Sköldheden, Jesper Vestlund, Christian E. Edvardsson, Elisabet Jerlhag

**Affiliations:** Institute of Neuroscience and Physiology, Department of Pharmacology, The Sahlgrenska Academy at the University of Gothenburg, Gothenburg, Sweden

**Keywords:** Addiction, Neuroscience

## Abstract

The underlying neurobiology of alcohol use disorder (AUD) is complex and needs further unraveling, with one of the key mechanisms being the gut-brain peptide ghrelin and its receptor (GHSR). However, additional substrates of the ghrelin pathway, such as liver-expressed antimicrobial peptide 2 (LEAP2), an endogenous GHSR inverse agonist, may contribute to this neurobiological framework. While LEAP2 modulates feeding and reward through central mechanisms, its effects on alcohol responses are unknown. The aim of the present study was therefore to identify the impact of central LEAP2 on the ability of alcohol to activate the mesolimbic dopamine system and to define its ability to control alcohol intake. These experiments revealed that central LEAP2 (i.e. into the third ventricle) prevented the ability of alcohol to cause locomotor stimulation in male mice, suppressed the memory of alcohol reward and attenuated the dopamine release in the nucleus accumbens caused by alcohol. Moreover, central LEAP2 reduced alcohol consumption in both male and female rats exposed to alcohol for 6 weeks before treatment. However, the serum levels of LEAP2 were similar between high- and low- alcohol-consuming (male) rats. Furthermore, central LEAP2 lowered the food intake in the alcohol-consuming male rats and reduced the body weight in the females. Collectively, the present study revealed that central LEAP2 mitigates alcohol-related responses in rodents, contributing to our understanding of the ghrelin pathway’s role in alcohol effects.

## Introduction

Alcohol use disorder (AUD) is a chronic disorder characterized by an individual’s inability to control or stop their alcohol consumption despite negative consequences. It has significant health, social, and occupational implications, making it a major public health concern worldwide. The acute effects of alcohol involve its rewarding properties which are mediated via a release of dopamine in the nucleus accumbens (NAc). Dopamine signaling in the NAc is also implicated in the manifestation of the disease, as evidenced by a lower number of dopamine D2 receptors in this region in individuals with AUD (for review see refs. [1, 2]). The neurobiology of AUD is intricate and warrants further investigation for a comprehensive understanding of the disease. One possible underlying mechanism for AUD involves the gut-brain peptide ghrelin and its receptor (growth hormone secretagogue receptor, GHSR), which are key for alcohol reward processing [3, 4].

While both ghrelin and the GHSR have been demonstrated to have a crucial role in feeding, their impact on reward processes has also been recognized [5, 6]. Specifically, ghrelin increases, whereas pharmacological suppression of GHSR attenuates reward-related behavior [7–13]. On a similar note, ghrelin infusion into the brain through the third ventricle, or reward-related areas such as the ventral tegmental area (VTA) and laterodorsal tegmental area (LDTg), elevates alcohol intake in rodents [7]. Contrarily, pharmacological suppression of central GHSR attenuates alcohol’s ability to activate the mesolimbic dopamine system, lowers alcohol intake, prevents relapse drinking, and attenuates the motivation to consume alcohol in rodents [7, 8, 14–21]

A more recently identified substrate of the ghrelin pathway is the liver-expressed antimicrobial peptide 2 (LEAP2), an endogenous inverse agonist on GHSR [22, 23]. While LEAP2 mainly is produced by the small intestine and liver [24, 25], its expression has been found in the brain, including the hypothalamus, midbrain, and hippocampus [26], and areas associated with reward such as NAc and VTA [27], albeit at relatively low expression compared to the small intestine and liver. Additionally, LEAP2 can be detected in human cerebrospinal fluid [28]. While ghrelin is associated with hunger and increases during low energy states, such as fasting, LEAP2 promotes satiety and increases after meal ingestion and during high energy states [22]. Thus, LEAP2 modulates feeding in an opposing manner to ghrelin and decreases the intake of chow [22], high-fat diet intake [29], and consumption of palatable foods [27]. Additionally, LEAP2 attenuates the ghrelin-induced food intake [26, 29].

Although, the exact mechanism by which LEAP2 affect food intake remains to be fully elucidated, peripheral LEAP2 is likely sensed by the hypothalamus to reduce hunger [30], whereas centrally expressed LEAP2 could directly affect the rewarding value of food through the central reward system [27]. Here, ghrelin and LEAP2 have differential effects on reward. As such, ghrelin induces reward-responses through activating accumbal dopamine signaling by itself and enhances food- and drug reward, including alcohol (for review see ref. [6]). Although LEAP2 has been less studied in this regard, LEAP2 attenuate the reward associated with high-preference foods, including dopaminergic signaling and rewarding memory [27, 29]. When it comes to reward responses, the central action of LEAP2 rather than peripheral appears to be important. As such, central, but not systemic, administration of LEAP2 reduces high-fat diet intake [29]. Similarly, recent data reveal that central, but not systemic administration of LEAP2 reduces the binge-like alcohol intake of a sweetened alcohol solution in mice [20]. In line with this, there appears to be no association between serum ghrelin levels and alcohol preference or intake in rats [8, 31], suggesting that the ghrelinergic system affects alcohol-related reward primarily through central mechanisms. To this end, additional studies are warranted to define the effects of central LEAP2 in alcohol-related responses in rodents. The present study therefore aims to identify the effects of central LEAP2 on alcohol-induced locomotor stimulation the memory of alcohol reward and alcohol-associated dopamine release in NAc in male mice. Moreover, the present study elucidates the possibility that central LEAP2 infusion reduces alcohol intake in male and female rodents. In attempts to explore the influence of circulating endogenous LEAP2 on alcohol intake, the serum levels of LEAP2 were measured in high- and low-alcohol-consuming rats of both sexes. Collectively, these experiments contribute to our understanding of the role of the ghrelin signaling pathway in alcohol-related responses.

## Material and methods

### Animals

For studies exploring the acute alcohol responses, adult male NMRI mice (25–30 g body weight at arrival; Charles River; Sulzfeld, Germany) were used. This strain was used as previous studies reveal that they respond robustly to alcohol [7, 32]. The mice were group-housed before surgery and thereafter held individually. They were kept in a room with a 12-h light-dark cycle (lights on at 7 am). Before the experiment, the mice were habituated to the test room for 60 min. For the intermittent access experiments, age-matched adult male and female Rcc/Han Wistar rats (approximately 300 g for males and 200 g for females at arrival; Envigo, Horst, Netherland) were used. This strain was selected as they consume high amounts of alcohol that give pharmacologically relevant blood alcohol concentrations [33]. Rats in the alcohol drinking experiment were maintained at a reversed light/dark cycle (lights off at 10 am). Both mice and rats had free access to water and standard chow (Teklad Rodent Diet; Envigo, Madison, WI, USA; 3 kcal/g innehåll), with a temperature of 20 °C and humidity of 50%. All animals were habituated to the animal facility one week before the experiment and were handled before experiment initiation. All experiments were conducted following guidelines from the Swedish Ethical Committee on Animal Research in Gothenburg (ethical permits: 1457/18, 3276/18, 1556/18, 3348/20) and every effort was made to maximize the animal’s well-being. When the experiments were finished both mice and rats were sacrificed, and the brains were collected. The brains were coronally cut to verify the placements of the guide and/or probe post-mortem. The placement was determined by the line left in the tissue by the injector and probe as previously published [27]. Only animals where correct placements could be confirmed were included in the statistical analysis (Supplementary Fig. 1a–d).

### Drugs

Alcohol (95% ethanol; Solveco, Stockholm, Sweden) was used. For behavioral and neurochemical experiments, alcohol (15% alcohol dissolved in vehicle (0.9% NaCl), 1.75 g/kg; 12.3 kcal/kg) was injected intraperitoneally (ip) five minutes before the start of the experiment as this design causes a robust alcohol response [7, 32]. For the intermittent access paradigm, alcohol was diluted in tap water to a final concentration of 20%. LEAP2 (LEAP-2 (38-77) (Human) / LEAP-2 (37-76) (Mouse), cat nr. 075-40 or LEAP-2 (37-76) (Rat), cat nr. 075-50; Phoenix Pharmaceuticals Inc., Burlingame, CA, USA) was dissolved in vehicle (Ringer solution; NaCl 140 mM, CaCl_2_ 1.2 mM, KCl 3 mM and MgCl_2_ 1 mM (Merck KGaA Darmstadt, Germany)). One hour before infusion into the third ventricle (icv) a dummy injector was inserted into the guide and then retracted to hamper spreading depression and remove clotted blood. The LEAP2 dose of 5.5μg in 1μl, infused 20 min before the test, was chosen based on a previous study, where LEAP2 had profound effects on reward with minimal effect on their normal behavior, as measured by locomotor activity parameters [27]. Further, a similar dose (5.0μg) has been shown to reduce alcohol intake in high consuming male and female mice [20]. LEAP2 or vehicle was infused at a volume of 1 μl over 60 s, the injector was left in place for another minute before being retracted to allow for complete diffusion of the drug. To enable LEAP2 infusion, a guide was inserted four days before the test, as previously described [34, 35]. Coordinates for the third ventricles of mice and rats are described in Supplementary Fig. 1.

### Locomotor activity

The ability of LEAP2 to block alcohol-induced locomotor stimulation in male mice (*n* = 42) was tested in open field arenas (42 × 42 × 20 cm; Med Associates Inc; Georgia, Vermont, USA), that are ventilated, and dim lit (4 lux). First, all mice were allowed to habituate to the open field arena for 60 min, after which LEAP2 or vehicle was infused 20 min before alcohol or vehicle. Creating the following treatment groups: vehicle-vehicle, LEAP2-vehicle, vehicle-alcohol, and LEAP2-alcohol. The activity (distance traveled in cm) was registered by infra-red beams located at the bottom of the open field boxes and it started 5 min following alcohol administration and was recorded for 60 min.

### Conditioned place preference

The ability of LEAP2 to block the memory of alcohol-induced reward (mCPP) in male mice (*n* = 16) was measured in the conditioned place preference (CPP) paradigm as previously described [36]. A two-chambered CPP apparatus (5 lux) with distinct tactile/visual cues was used. The paradigm consists of pre-conditioning (day 1), conditioning (days 2–5), and post-conditioning (day 6), where each session was 20 min. The mice were allowed to freely explore both compartments at preconditioning. Alcohol was then paired with the least preferred compartment, whereas the preferred chamber was paired with vehicle, during the conditioning days in a biased design. On post-conditioning day, LEAP2 or vehicle was infused 20 min before free exploration of both compartments. Creating the following treatment groups: alcohol-vehicle, alcohol-LEAP2. In this context, as treatment was administered on the post-conditioning day, the retrieval of the rewarding memory was measured in a memory CPP (mCPP). The mCPP was calculated as the difference of the total time spent in the alcohol-paired compartment during the post-conditioning and pre-conditioning sessions as a percent of the total time.

### In vivo microdialysis and dopamine measurements

The ability of LEAP2 to affect the alcohol-induced dopamine release in NAc shell (NAcS) of male mice was measured using in vivo microdialysis studies. Two days prior to the microdialysis experiment, male mice (*n* = 20) underwent surgery as previously described [37]. During surgery, a guide aiming at third ventricle and a probe (20 kDa cut off with a 1 mm exposed length, HOSPAL, Gambro, Lund, Sweden) targeting NAcS was inserted (Supplementary Fig. 1b). The probe was connected to a pump and perfused with Ringer’s solution at a rate of 1.6 μl/min and a two-hour habituation period was performed before initiation of the experiment. Samples was thereafter collected every 20 min throughout the experiment. After a baseline (−80 to −60 min), LEAP2 or vehicle was infused (at −20 min). 20 min later alcohol was injected (at 0 min) and an additional eight samples were collected (20–160 min). Control experiments previously showed that LEAP2 does not alter dopamine in NAcS per se [27]. The dopamine content in each sample was measured using a high-performance liquid chromatography system with electrochemical detection as described previously [37, 38]. The dopamine levels were calculated as a percentage of the mean of the three baseline values before LEAP2/vehicle treatment.

### Alcohol intake experiments

The potential of LEAP2 to reduce alcohol intake in male (*n* = 10) and female (*n* = 12) rats was tested in the intermittent access paradigm. This paradigm has previously been validated to induce robust levels of voluntary alcohol consumption in rats [33], and has been used extensively in our lab to study AUD [31, 37]. In this paradigm, the rats have access to alcohol (20%) and water at three 24-h sessions per week (Monday, Wednesday, and Friday), whereas only water was provided on the other days. The rats consumed alcohol for 6 weeks (baseline drinking) before treatment, which took place at two alcohol-drinking sessions with each rat serving as its own control in a counterbalanced within-subjects design. At alcohol drinking session 1, LEAP2 or vehicle was infused 20 min prior to the lights went out and rats received their alcohol bottle. At alcohol drinking session 2, the rats were infused with the opposite treatment allowing for paired comparisons. Importantly, the intake of vehicle-treated rats was similar independent of treatment day and similar to that during baseline. At each treatment day, the intake of alcohol, water, and food was measured 2-, 4- and 24 h after LEAP2 or vehicle infusion. Additionally, alcohol preference (alcohol intake over total intake presented as percent) and total intake (alcohol and water together) were calculated for each time point. The body weight change (weight after treatment compared to weight before treatment) was measured.

In a separate intermittent access experiment, the effects of long-term alcohol consumption on the serum levels of LEAP2 were evaluated in rats of both sexes. Male (*n* = 24) and female (*n* = 24) rats were allowed to drink alcohol for 10 weeks and were euthanized at the end of an alcohol drinking session and trunk blood was collected into serum tubes (10 mL *95 ×16.8* *mm Z-gel*, Sarstedt, Helsingborg, Sweden). The serum tubes were shaken and incubated for at least 30 min in line with the manufacturer’s instructions. After centrifugation (4600 rpm, 10 min, RT) the serum concentration of LEAP2 was measured by ELISA kits according to the manufacturer’s instructions (MBS7229156, MyBioSource, San Diego, CA, USA). A microplate photometer (Multiskan GO, Thermo Fisher Scientific, Darmstadt, Germany) at a defined wavelength (450 nm) was used to detect the fluorescence intensity of each duplicate serum sample placed on multi-well plate. Here, circulating levels of LEAP2 could only be reliably detected in males. For all females, the levels were below the limit of detection.

### Statistics

A one-way ANOVA with Bonferroni post-hoc tests analyzed the locomotor activity data. A two-tailed unpaired t-test analyzed the mCPP data and serum levels of LEAP2. The microdialysis experiments were evaluated by a repeated two-way ANOVA followed by Bonferroni post-hoc test for comparisons between different treatments and specifically at given time points. The data from the alcohol drinking experiment were analyzed by a two-tailed paired t-test. Correlation between alcohol consumption and serum LEAP2 was analyzed using Pearson correlation. Data are presented as mean ± SEM. A probability value of *P* < 0.05 was considered as statistically significant and multiple corrections were used. GraphPad Prism® 10.1.1. (GraphPad Software, Inc. La Jolla, CA, USA) was used for all statistical calculations.

## Results

### LEAP2 attenuates the ability of alcohol to induce locomotor stimulation, rewarding memory, and release of dopamine in NAcS in male mice

**Fig. 1:**
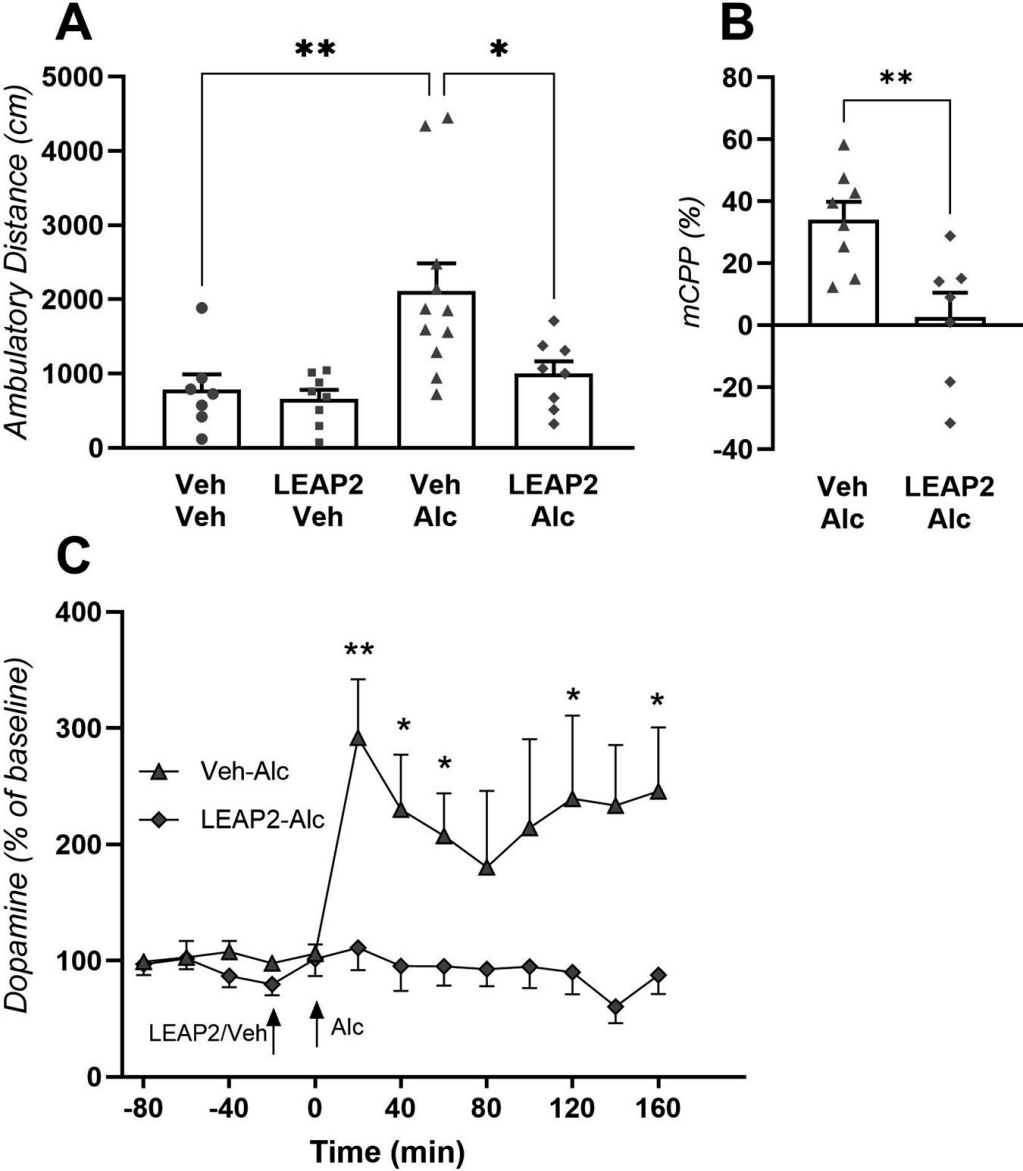
Central LEAP2 reduces alcohol-induced behavior and neurochemistry in male mice. **A** In locomotor activity tests of male mice, alcohol causes a locomotor stimulation and central LEAP2 blocks this effect (*n* = 7–11, one-way ANOVA). **B** Furthermore, in male mice central infusion of LEAP2 suppresses the memory of alcohol reward in the conditioned place preference paradigm (mCPP, *n* = 7–8, unpaired t-test). **C** The alcohol-induced release of dopamine in nuclease accumbens is attenuated by central LEAP2 infusion in male mice (*n* = 8-9, repeated measures two-way ANOVA). Data is shown as mean±SEM. **P < 0.01, *P < 0.05.

There was an overall effect on locomotor activity in male mice treated with LEAP2 and alcohol (F_3,30_ = 6.17, *P* = 0.0013; Fig. 1a). LEAP2 blocked (*P* = 0.0223, *n* = 8) the alcohol-induced locomotor stimulation (*P* = 0.0071, *n* = 11) in male mice. This is further evident as the activity between vehicle (*n* = 7) and LEAP2-alcohol-treated mice are similar (*P* = 0.8742). LEAP2 did not influence the activity per se (*P* = 0.9973, *n* = 8). In total 8 mice were excluded due to misplaced infusion sites. Next, LEAP2’s effect on the memory of alcohol reward was evaluated using the mCPP paradigm. As expected, LEAP2 attenuated the memory of alcohol reward (Fig. 1b, *n* = 7) compared to vehicle (*n* = 8, t_13_ = 3,31, *P* = 0.0057). One mouse was excluded due to a misplaced infusion site.

Further support for LEAP2’s ability to attenuate the stimulatory and rewarding properties of alcohol is provided by the in vivo microdialysis studies in which LEAP2 suppresses the ability of alcohol to cause a release of dopamine (Fig. 1c). Supportively, there was an overall effect of treatment (F_1,15_ = 14.70, *P* = 0.0016), time (F_12,180_ = 2.89, *P* = 0.0011), and interaction (F_12,180_ = 2.91, *P* = 0.0011). The dopamine levels were lower in LEAP2-alcohol-treated mice (*n* = 7) compared to those treated with vehicle-alcohol (*n* = 8) at 20 (*P* < 0.01), 40-60 (*P* < 0.05), and 140-160 (*P* < 0.05) min. Three mice were excluded due to misplaced infusion sites/probe placements.

### LEAP2 reduces alcohol intake in male and female rats

**Fig. 2:**
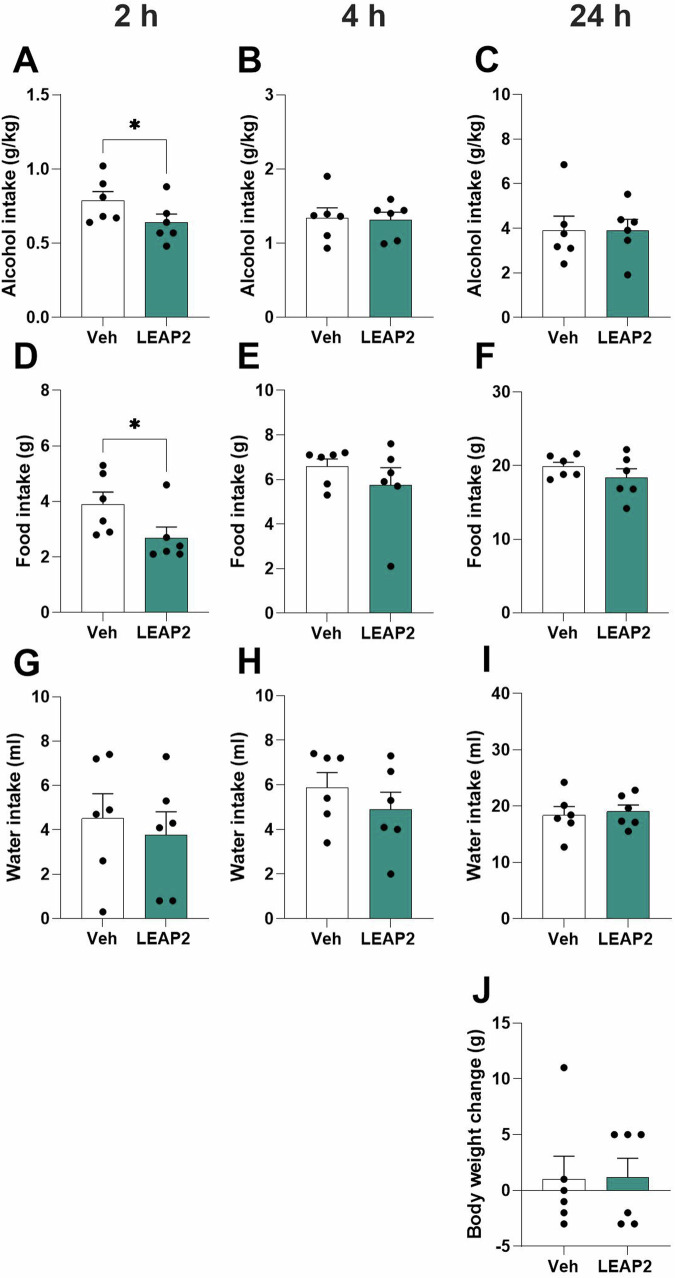
Central LEAP2 decreases alcohol intake in male rats. Compared to vehicle, central LEAP2 infusion (**A**) reduces the alcohol intake at the 2-h timepoint, whereas no treatment effect is observed at (**B**) 4-h or (**C**) 24-h timepoints in male rats. Additionally, LEAP2 (**D**) decreases food intake at the 2-h but not at (**E**) 4-h or (**F**) 24-h timepoint. As further evident in male rats, LEAP2 does not alter the water intake at any measured time point (**G**–**I**), (**J**) or affects the body weight. Data is shown as mean±SEM (*n* = 6, paired t-test), *P < 0.05.

**Fig. 3:**
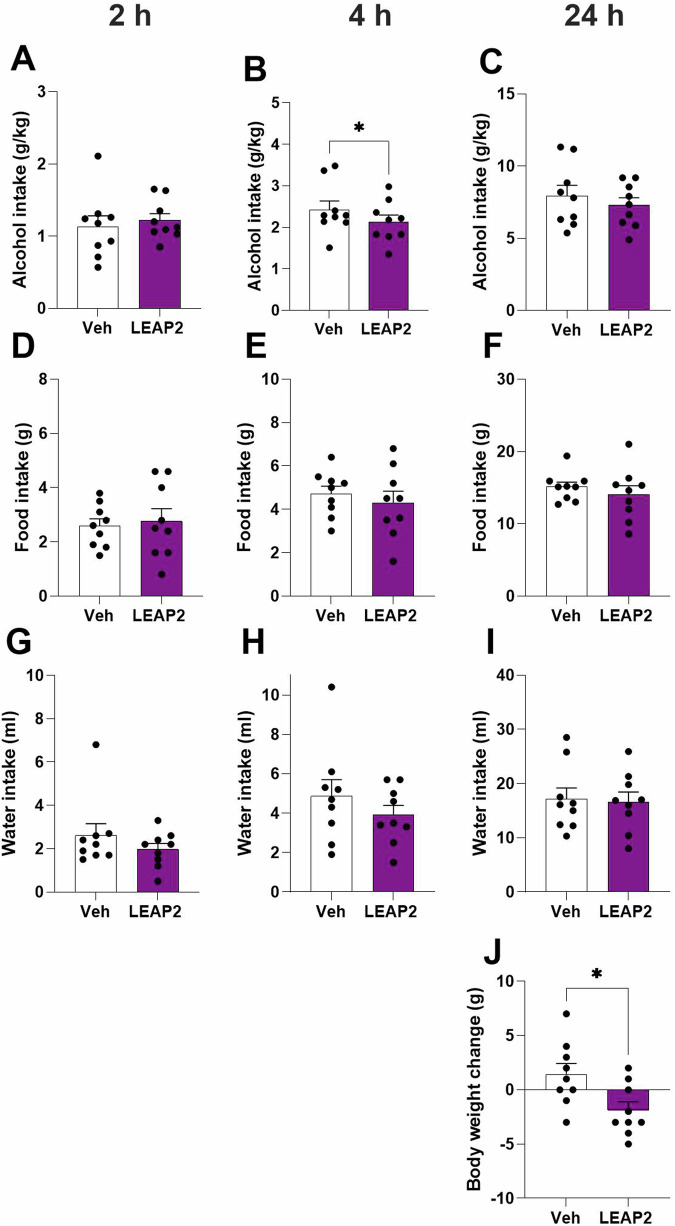
Central LEAP2 reduces alcohol intake in female rats. Central LEAP2 infusion into female rats (**A**) does not alter the alcohol intake at the 2-h timepoint, (**B**) whereas it decreases alcohol intake at the 4-h timepoint. **C** LEAP2-treated female rats consumed as much alcohol as vehicle-treated at the 24-h timepoint. In females, LEAP2 does neither alter the (**D**–**I**) food intake nor (**G**–**I**) water intake at any measured time point. **J** Additionally, compared to vehicle LEAP2 suppresses the body weight in female rats. Data is shown as mean±SEM (*n* = 9, paired t-test), *P < 0.05.

Additional evidence for the role of LEAP2 in alcohol responses was provided by alcohol-drinking experiments in male (Fig. 2, *n* = 6) and female rats (Fig. 3, *n* = 9), where each rat got both treatments. Four males and three females were excluded due to infusion misplacements.

Compared to vehicle, LEAP2 reduced the alcohol intake at the 2-h time point in male rats (t_5_ = 3.36, *P* = 0.0202, Fig. 2a), exposed to alcohol for 6 weeks before treatment (4.3±0.5 g/kg). However, LEAP2 did not influence alcohol intake at 4- (t_5_ = 0.19, *P* = 0.8579, Fig. 2b) or 24-h (t_5_ = 0.00, *P* = 0.9966, Fig. 2c) timepoints. Additionally, the food intake was lowered after LEAP2 at the 2-h timepoint (t_5_ = 3.05, *P* = 0.0286, Fig. 2d). Similar to alcohol, LEAP2 did not alter food intake at the 4- (t_5_ = 1.26, *P* = 0.2641, Fig. 2e) or 24- (t_5_ = 1.83, *P* = 0.1275, Fig. 2f) hour timepoints or alter the body weight change (Fig. 2j, t_5_ = 0.05, *P* = 0.9603). Moreover, LEAP2 did not alter the water intake at any investigated timepoints (2-h, Fig. 2g, t_5_ = 0.39, *P* = 0.7099; 4-h, Fig. 2h, t5 = 0.90, *P* = 0.4075; 24-h, Fig. 2i, t5 = 0.64, *P* = 0.5523), the preference for alcohol (2-h, t_5_ = 0.60, *P* = 0.5719; 4-h, t_5_ = 0.73, *P* = 0.5005; 24-h, t_5_ = 0.35, *P* = 0.7409) or total fluid intake (2-h, t_5_ = 0.2.04, *P* = 0.0971; 4-h, t_5_ = 1.01, *P* = 0.3609; 24-h, t_5_ = 0.67, *P* = 0.5307, Supplementary Fig. 1a–f).

**Fig. 4:**
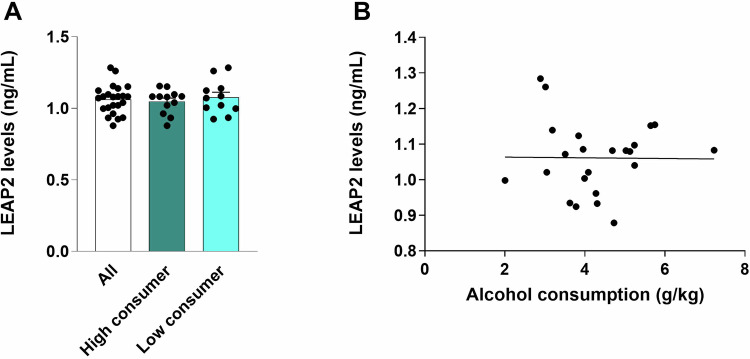
Serum LEAP2 levels are not associated with alcohol consumption in male rats. Circulating levels of LEAP2 in serum could only be detected in males. **A** There was no difference between high (>3.5 g/kg) and low alcohol consumers (<3.5 g/kg). **B** Further, there was no evident correlation between alcohol consumption and LEAP2 serum levels. Data is shown as mean±SEM (*n* = 8-9 in each group, unpaired t-test and Pearson correlation).

Circulating LEAP2 levels could only be reliably detected in males (*n* = 23, 1.06 ± 0.02 ng/ml, Fig. 4). While the above data revealed that central LEAP2 lowers alcohol intake in male rats, circulating LEAP2 did not correlate to alcohol drinking in a separate cohort of male rats. Specifically, the LEAP2 levels in serum were similar in high (>3.5 g/kg, *n* = 12, 1.05 ± 0.02 ng/ml) and low (<3.5 g/kg, *n* = 11, 1.08 ± 0.04 ng/ml) alcohol-consuming rats (t_21_ = 0.70, *P* = 0.493, Fig. 4a). Further, there was no correlation between alcohol consumption and circulating LEAP2 levels (*p* = 0.960, Fig. 4b). One serum sample from male rats was excluded due to contamination which disrupted the absorbance reading from the ELISA.

While LEAP2 did not alter the alcohol intake at the 2-h timepoint (t_8_ = 0.65, *P* = 0.5359, Fig. 3a) in female rats under the same conditions (5.9 ± 0.6 g/kg), it reduced it at the 4-h timepoint (t_8_ = 3.36, *P* = 0.0100, Fig. 3b). Additionally, LEAP2 did not influence alcohol intake at the 24-h (t_8_ = 1.11, *P* = 0.3011, Fig. 3c) timepoint. Further, LEAP2 did not change the food intake at the 2- (t_8_ = 0.40, *P* = 0.6994, Fig. 3d), 4- (t_8_ = 1.06, *P* = 0.3308, Fig. 3e) or 24- (t_8_ = 1.10, *P* = 0.3052, Fig. 3f) hour timepoints. Neither did LEAP2 influence the water intake at any investigated timepoints (2-h, Fig. 3g, t_8_ = 0.98, *P* = 0.3561; 4-h, Fig. 3h, t8 = 1.41, *P* = 0.1970; 24-h, Fig. 3i, t8 = 0.36, *P* = 0.7316). However, LEAP2 reduced the body weight change (Fig. 3j, t_8_ = 2.58, *P* = 0.0325). LEAP2 treatment did not alter the preference for alcohol (2-h, t_8_ = 1.54, *P* = 0.1620; 4-h, t_8_ = 0.45, *P* = 0.6656; 24-h, t_8_ = 0.58, *P* = 0.5802) or total fluid intake (2-h, t_8_ = 0.84, *P* = 0.4242; 4-h, t_8_ = 2.16, *P* = 0.0627; 24-h, t_8_ = 0.77, *P* = 0.4637, Supplementary Fig. 2g–l).

In a separate set of alcohol-consuming female rats, LEAP2 was measured in serum. However, LEAP2 was not detected in serum from female rats due to concentrations below the detection limit.

In male rats, central LEAP2 infusion lowered the total caloric intake at the 2-h time point (t_5_ = 3.13, *P* = 0.0259; Supplementary Fig. 3a), while it did not influence it at 4- or 24-h time points (t_5_ = 1.09, *P* = 0.3260 and t_5_ = 1.28, *P* = 0.2571; Supplementary Fig. 3b, c). In female rats, the caloric intake is similar in rats treated with vehicle or LEAP2 (t_8_ = 0.41, *P* = 0.6897; t_8_ = 1.36, *P* = 0.2123; t_8_ = 1.35, *P* = 0.2131; Supplementary Fig. 3d–f).

## Discussion

The present study contributes towards the further understanding of central effects influenced by LEAP2, as it modulates alcohol responses in rodents.

Male mice exposed to alcohol display a locomotor stimulation and dopamine release in NAcS, effects suppressed by central infusion of LEAP2. As these parameters reflect alcohol’s rewarding properties [6], a tentative explanation for the present findings is that LEAP2 attenuates alcohol-induced reward. Supportively, we recently showed that the infusion of central LEAP2 reduces the intake of palatable food as well as decreases the accumbal dopamine release associated with palatable food [27]. Similarly, it has been shown that binge eating of high-fat diet, also considered rewarding, is lowered after central LEAP2 infusion in mice [29]. Further, the synthetic GHSR antagonist JMV2959 or genetic deletion of GHSR have previously been shown to attenuate alcohol-induced locomotor activity and accumbal dopamine release [7, 39, 40], in agreement with the present LEAP2 results.

While suppressed alcohol-induced reward may contribute to LEAP2s ability to lower alcohol intake, other factors may influence this treatment outcome. Indeed, the memory of alcohol reward is another factor influencing alcohol consumption [2] and another factor here suppressed by central LEAP2 infusion. Supportively, central LEAP2 suppresses the memory of palatable foods, and the attenuation is influenced by the rewarding component of the food [27]. An effect of ghrelin singling on memory function is further supported as ghrelin stimulates hippocampal circuits [41–43]. Comparably to LEAP2, GHSR antagonist JMV2959 and inverse agonist BIM28163, as well as genetic deletion of GHSR, reduces alcohol-associated rewarding memory [7, 15]. Together this suggests that that LEAP2 modulates the memory related to reward, an important aspect of addiction. It should however be noted that only male mice were included in these behavioral and neurochemical studies as these models are less established in females, and this should be considered a limitation.

The present study further demonstrated that central infusion of LEAP2 reduced the intake of alcohol in both male and female rats. This is in line with a recent study in which the binge drinking of alcohol is lowered after central infusion of LEAP2 in male and female mice consuming a sweetened alcohol solution [20]. In the present study, the decrease in alcohol consumption is noticeable at the 2-h timepoint in males, while it is observed at the 4-h timepoint in females. Supportively, both acute and repeated pharmacological blockade using synthetic GHSR antagonists lowers alcohol consumption in male rodents [7, 14, 15, 17, 18, 39] and the effect is comparable to that of a the GHSR synthetic antagonist BIM28163 [7]. Similarly, genetic deletion of GHSR attenuates binge-like alcohol consumption in rats [21].

We however did not find any correlations between serum levels of LEAP2 and alcohol intake in male rats. Similarly, central, but not peripheral administration of LEAP2 reduces binge drinking of a sweetened alcohol solution in male and female mice [20]. This is in line with the notion that the peripheral ghrelinergic pathway is not involved in mediating alcohol intake. As such, we have previously shown that there is no difference in serum ghrelin levels between alcohol preferring and non-preferring rats [8] and neutralizing endogenous peripheral ghrelin does not affect alcohol intake in rats [31]. Taken together these data imply that LEAP2 may act via central rather than peripheral mechanisms to modulate alcohol-related responses.

Supportively, LEAP2 is both expressed centrally and can be detected in cerebrospinal fluid [26, 28]. LEAP2s potential mechanisms of action may involve areas associated with reward processing, given its expression in brain regions crucial for reward, such as the NAc and LDTg [27]. While this remains to be explored with regards to alcohol, it is applicable for feeding since the local infusion of LEAP2 into the LDTg, the area with the highest LEAP2 expression, reduced the intake of palatable food [27]. Supportively, ghrelin acts via reward areas like LDTg to control alcohol intake, reward and the consumption of food [7, 11, 44]. Further, LEAP2 has been shown to directly influence dopamine receptors through GHSR heterodimer complexes, which might reduce dopamine signaling directly through presynaptic and somatodendritic dopamine feedback [45, 46].

LEAP2 resulted in decreased food intake specifically at the 2-h timepoint in male rats, with no apparent effect on food intake at any other time points in males, nor on feeding behavior in females at any time. Supportively, it has been demonstrated that LEAP2 decreases the consumption of chow [22], high-fat diet [29], or palatable food [27] in male rodents. Additionally, previous studies found that LEAP2 attenuates the ghrelin-induced food intake [26, 29] and conversely, ghrelin enhances chow and palatable food intake [13, 47, 48]. This study did not define the mechanisms behind the reduction in chow intake in males, but it is plausible that homeostatic effects, known to be mediated by the ghrelin pathway, could be involved [5]. Although LEAP2 did not alter food intake in females, this study enhances our comprehension of central LEAP2’s impact on female feeding, as this aspect has not been previously investigated. The sex-specific effects of LEAP2 on food intake extend to body weight, as evidenced by its reduction in female rats while having no discernible impact on male rats. The mechanisms for this decline in body weight in females remain to be explored but may involve a reduced caloric intake. However, this appears less likely as LEAP2 does neither affect the total caloric intake or chow intake in females. Potentially, a combination of reduced, albeit insignificant, caloric intake and total fluid intake at 24 h could result in the observed weight reduction. It may also involve enhanced energy expenditure or reduced adiposity which both are influenced by LEAP2 and synthetic GHSR antagonists [49, 50].

As summarized above, LEAP2 has some sex-diverging effects in these alcohol-drinking rats. Further, LEAP2 was not detected in serum from female rats, which highlights potential sex differences in endogenous levels of LEAP2. Supportively, previous studies have revealed that expression of LEAP2 differs between sex, although its functional impact needs further investigation [25, 51]. The observed sex-diverging effects in the present study may be attributed to pharmacokinetic or hormonal differences between sexes. However, the exact mechanisms need to be elucidated in the future. The sex-divergent effect of LEAP2 is not surprising, as sex differences in ghrelin responses are apparent in relation to both feeding behaviors and for example anxiety-like behavior [52, 53].

Although a suppressive effect of LEAP2 on alcohol-related responses was observed, the present study has several limitations. Although we assessed drinking behaviors in both male and female rats, the effect of LEAP2 on alcohol-mediated reward was only measured in male mice, which limits the interpretation of LEAP2s effect. Further, we focused here on the central effects of LEAP2, not accounting for possible systemic effects which may influence the results. However, a recent study showed that peripheral LEAP2 had no apparent effect on alcohol consumption [20]. Another factor that might influence the treatment outcome is a reduced dopaminergic tone in NAc or altered locomotor behavior at baseline. However, this appears less likely as LEAP2 neither affects dopamine in NAc nor motor behavior per se [27].

Regarding LEAP2s therapeutic potential for AUD, even though the effect on alcohol consumption was significant, the reduction was minor. This is likely attributed to the short half-life of LEAP2 (about 9 min) [54] in relation to the relatively long period of measurement (hours). As such, the direct therapeutic potential of exogenous LEAP2 in AUD is limited, although our results would suggest that brain penetrable GHSR inverse agonists or emerging long-acting LEAP2 analogs [55] may be a viable treatment for AUD. However, as an endogenous substance that is highly affected by for example diet [27], energy status [22, 56] and microbiota [25], LEAP2 further increases our understanding of the association of physiological status and AUD through the ghrelinergic system. Here, elevating LEAP2 indirectly through dietary and microbiota interventions may prove valuable for sustainable long-term treatment of AUD, perhaps particularly in preventing relapse, due to LEAP2s strong effects in attenuating the rewarding effect and rewarding memory of alcohol. It should be noted, that although the difference between peripheral and central LEAP2 effects is somewhat unclear, we recently showed that dietary changes can have profound effects on LEAP2 expression in the brain, in particular those associated with reward [27]. Lastly, it should be mentioned that LEAP2 potentially may induce for example anhedonia, given its effect on reducing dopaminergic reward responses.

Conclusively, the present study demonstrates that central LEAP2 suppresses alcohol-related responses and thereby contributes to the characterization of the ghrelin pathway’s role in AUD.

## Supplementary information

Tufvesson-Alm et al. Supplementary Material

## Data Availability

Data will be made available on request.
