# Supplementary material for: LEAP2, a ghrelin receptor inverse agonist, and its effect on alcohol-related responses in rodents
Source: Transl Psychiatry. 2024 Oct 2;14:401. doi: 10.1038/s41398-024-03136-y (PMC11446955; doi:10.1038/s41398-024-03136-y)
Supplement: Supplementary file 1 — Tufvesson-Alm et al. Supplementary Material [file 41398_2024_3136_MOESM1_ESM.pptx]

## Slide 1
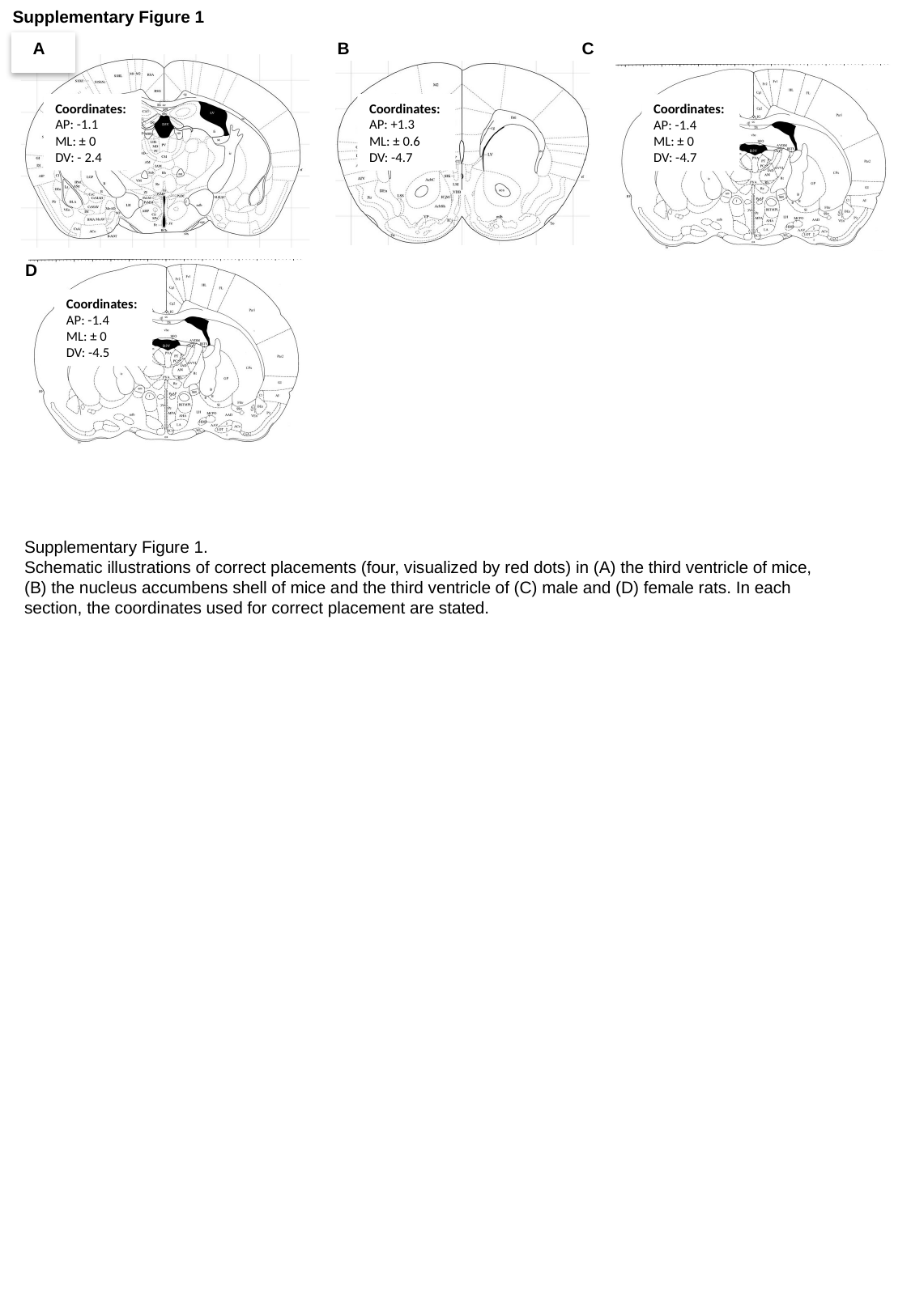

Supplementary Figure 1
A B C
Coordinates:
AP: -1.1
ML: ± 0
DV: - 2.4
Coordinates:
AP: +1.3
ML: ± 0.6
DV: -4.7
Coordinates:
AP: -1.4
ML: ± 0
DV: -4.7
D
Coordinates:
AP: -1.4
ML: ± 0
DV: -4.5
Supplementary Figure 1.
Schematic illustrations of correct placements (four, visualized by red dots) in (A) the third ventricle of mice, (B) the nucleus accumbens shell of mice and the third ventricle of (C) male and (D) female rats. In each section, the coordinates used for correct placement are stated.

## Slide 2
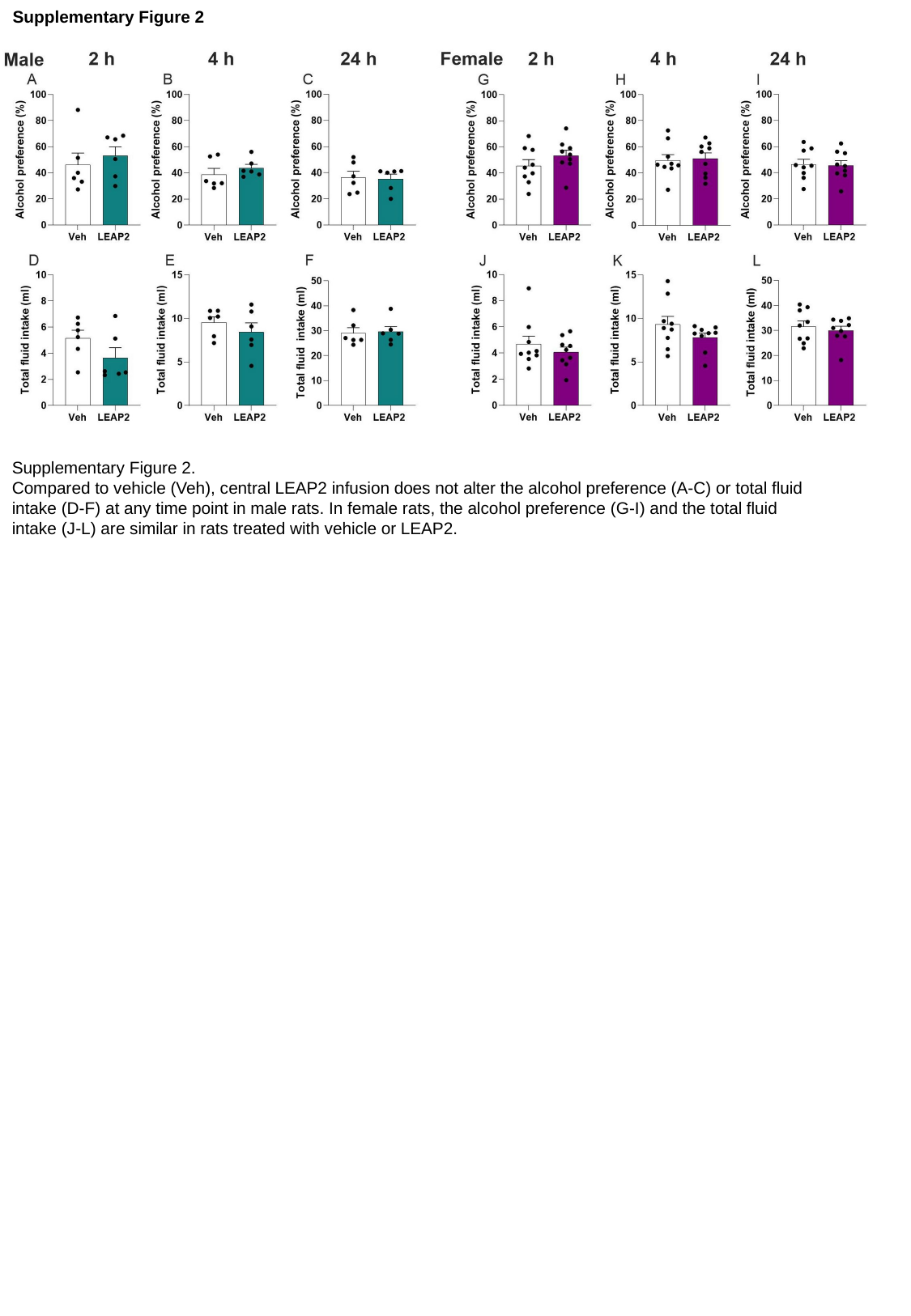

Supplementary Figure 2
Supplementary Figure 2.
Compared to vehicle (Veh), central LEAP2 infusion does not alter the alcohol preference (A-C) or total fluid intake (D-F) at any time point in male rats. In female rats, the alcohol preference (G-I) and the total fluid intake (J-L) are similar in rats treated with vehicle or LEAP2.

## Slide 3
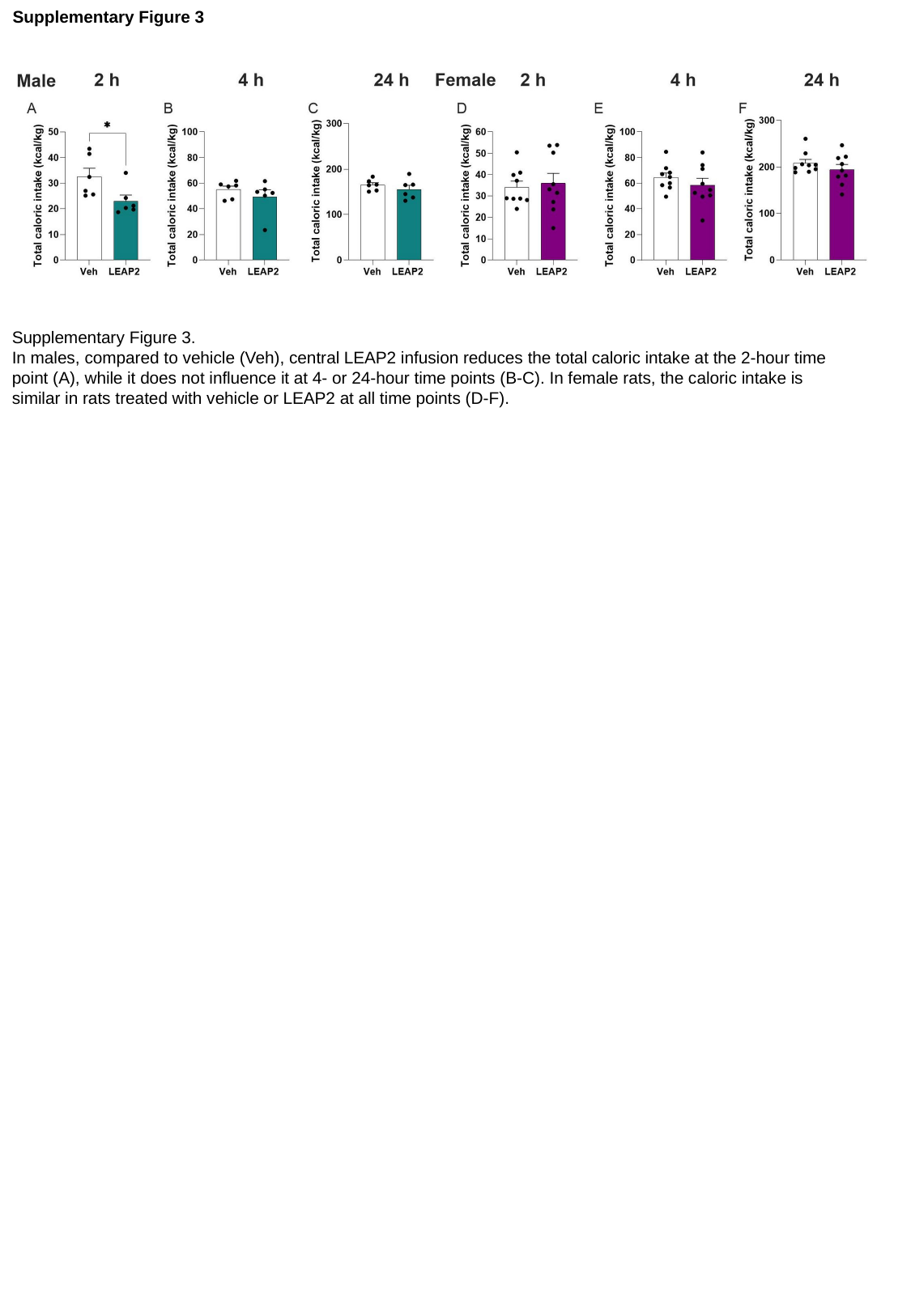

Supplementary Figure 3
Supplementary Figure 3.
In males, compared to vehicle (Veh), central LEAP2 infusion reduces the total caloric intake at the 2-hour time point (A), while it does not influence it at 4- or 24-hour time points (B-C). In female rats, the caloric intake is similar in rats treated with vehicle or LEAP2 at all time points (D-F).
